# Fatal Kikuchi-like lymphadenitis associated with connective tissue disease: a report of two cases and review of the literature

**DOI:** 10.1186/s40064-015-0925-7

**Published:** 2015-04-08

**Authors:** Vijay Sharma, Rosslyn Rankin

**Affiliations:** Department of Pathology, Raigmore Hospital, Inverness, Scotland; Department of Pathology, Aberdeen Royal Infirmary, Foresterhill Aberdeen, Scotland; Division of Applied Medicine, School of Medicine and Dentistry, University of Aberdeen, Aberdeen, Scotland

**Keywords:** Kikuchi-Fujimoto disease, Necrotising lymphadenitis, Connective tissue disease, Systemic lupus erythematosus, Lymphadenopathy

## Abstract

**Introduction:**

Kikuchi-Fujimoto disease, is usually a benign self-limiting disease which typically affects young females under the age of 30 years and resolves without treatment within six months. However, when it occurs in the context of connective tissue disease, particularly systemic lupus erythematosus (SLE), it is usually associated with a flare-up of the patient’s symptoms, requiring treatment, and can lead to severe, potentially life-threatening sequelae.

**Case description:**

Here, we report and compare two cases of unclassifiable connective tissue disease who developed a Kikuchi-like lymphadenitis and sepsis-like clinical syndrome, including disseminated intravascular coagulation, which proved rapidly fatal.

**Discussion and evaluation:**

In our review of the literature, we found 55 cases of Kikuchi-Fujimoto disease occurring in the context of definite connective tissue disease, 50 of which were associated with SLE. Of the 55 cases, 22 (40%) had simultaneous onset with, 19 (35%) predated the onset of and 14 (25%) developed after the associated connective tissue disease. Life-threatening autoimmune sequelae were reported in 8 cases, 2 of which were fatal. The aetiology of the association remains unknown.

**Conclusion:**

Kikuchi-Fujimoto disease is a histopathological diagnosis, and although the classical form appears to represent a distinct entity, it is unclear whether it is always the same entity, regardless of the context in which it occurs, or whether it represents a histological pattern with a variety of possible causes. In any case, the possibility of auto-immune sequelae in patients with known autoimmune disease should always be considered if these patients present with a sepsis-like clinical syndrome and no infective source is identified.

## Introduction

Kikuchi-Fujimoto disease, also known as histiocytic necrotising lymphadenitis, is a benign self-limiting disease which typically affects young females under the age of 30 years and resolves without treatment within six months. The disease was originally described in Japan in 1972 and is known to be more common in Asian populations. The aetiology is unknown, and knowledge about the condition is derived mostly from case reports. Although both viral and autoimmune aetiologies have been proposed, there is no convincing evidence linking Kikuchi-Fujimoto disease to either of these aetiologies. Although the disease course is normally self-limiting, fatal cases are known to occur, with a reported fatality rate of 2.1% (Kucukardali et al. 2007; Chan et al. 1989). An association between a Kikuchi-like lymphadenitis and autoimmune disease, particularly systemic lupus erythematosus (SLE), has been noted. The lymphadenitis occurring in this context can produce a sepsis-like clinical syndrome which is potentially life-threatening. Here, we compare and contrast two cases of patients with longstanding connective tissue disease which proved hard to classify, both of whom developed a fatal Kikuchi-like lymphadenitis.

### Case 1

This was a case of a 57 year old woman with a long history of connective tissue disease. She suffered from Raynaud’s phenomenon for 35 years, and, at the age of 29, a biopsy of nodular lesions affecting the elbows, ankles and hands revealed rheumatoid nodules/granuloma annulare. She subsequently developed widespread inflammatory arthritis. She also suffered from psoriasis, and psoriatic arthropathy was the preferred diagnosis for a period of time. However, she had an autoantibody profile which included a positive ANF with a speckled pattern, positive anti-La and positive RNP, and mixed connective tissue disease was eventually considered to be the most likely diagnosis. Rheumatoid factor was negative. She had multiple drug allergies, including to numerous antibiotics, and declined systemic therapy. Her arthritis was treated with steroid injections.

She was admitted to the Medical Unit with a few days history of fever, right flank pain and mild urinary frequency. She had been having recurrent urinary tract infections over the previous three months and was diagnosed with pyelonephritis. She was treated with teicoplanin and gentamicin and her symptoms improved. However, blood cultures were negative and a renal ultrasound scan was normal. She was discharged home, but was readmitted three days later with a recurrence of her symptoms. On admission she had a fever of 38-40°C and an erythematous rash on her trunk, but physical examination was otherwise unremarkable. Her CRP increased from 17 on admission to 72 the following morning. Her white cell count was not elevated and a chest X-ray showed no obvious consolidation. Her antibiotics were restarted. Three days later, she rapidly became breathless, hypotensive, poorly perfused and tachycardic. Blood analyses revealed significant biochemical and haematological derangement including raised phosphate and creatinine levels, low albumin, a neutrophilia and thrombocytopaenia/disseminated intravascular coagulation (DIC) (Table 1). A diagnosis of septic shock was made. During transfer to intensive care, she suffered a cardiac arrest and failed to respond to resuscitation attempts.

**Table 1:** **Comparison of the biochemical and haematological parameters of both cases following the onset of their deterioration into thrombocytopenia/DIC**

	**Case 1**	**Case 2**	**Normal range**
**Sodium (mmol/l)**	131	138	136-144
**Potassium (mmol/l)**	6.3	5.2	3.8-5.0
**Chloride (mmol/l)**	104	110	95-105
**Bicarbonate (mmol/l)**	6	7	24-32
**Urea (mmol/l)**	8.1	6.9	2.5-6.0
**Creatinine (μmol/l)**	234	84	45-100
**Estimated GFR (ml/min/1.73 m** ^**2**^ **)**	20	>60	>90*
**Lactate (mmol/l)**	NA	1.32	0.63-2.44
**Bilirubin (μmol/l)**	7	14	<21
**Alkaline Phosphatase (units/l)**	101	86	30-90
**ALT (units/l)**	399	1514	5-40
**Albumin (g/l)**	11	15	36-52
**Calcium (mmol/l)**	1.54 2.12 adjusted	2.13 2.63 adjusted	2.10-2.60
**Magnesium (mmol/l)**	1.16	1.01	0.7-1.0
**Phosphate (mmol/l)**	3.97	1.93	0.80-1.50
**C-reactive protein (mg/l)**	72	18	0-9
**Troponin I (μg/l)**	4.66	NA	0.01-0.04
**Haemoglobin (g/l)**	179	88	118-148
**Haematocrit**	0.550	0.261	0.36-0.44
**Platelets (10** ^**9**^ **/l)**	27	13	150-400
**White cell count (10** ^**9**^ **/l)**	22.8	4.1	4.0-11.0
**Neutrophils (10** ^**9**^ **/l)**	17.7	1.4	2.0-7.5
**Prothrombin time (seconds)**	Too prolonged to quantify	46	10-14
**Partial prothrombin time (seconds)**	Too prolonged to quantify.	91	26-36
**Fibrinogen (g/l)**	1.2	0.6	1.5-4.0
**D-Dimer (ng/ml)**	34761	NA	<250

*Although this is the normal range, eGFR is not accurate at near-normal levels of GFR and many laboratories, including ours, do not quantify the eGFR if it is above 60.

**Figure 1:**
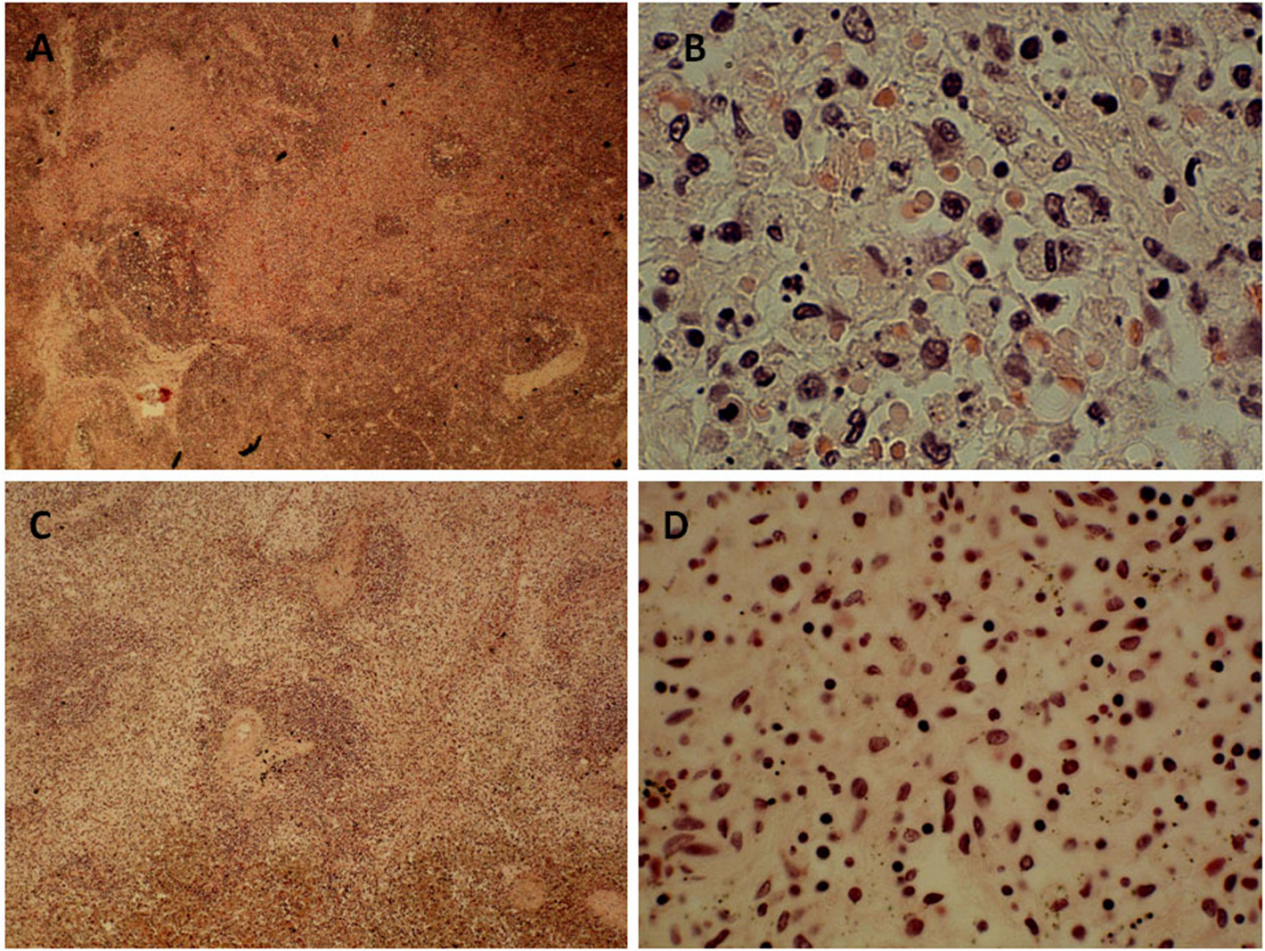
**Histology of lymph nodes and spleen from Case 1. A-B**: Low (x100) and high (x400) power views of peritonsillar lymph node. **C-D**: Low (x100) and high (x400) power views of spleen.

Post mortem examination was unremarkable, and no infective source was identified. Histology of lymph nodes harvested from the neck bilaterally, peritonsillar area, tracheobronchial region, axillae, groins and small bowel mesentery, as well as the spleen, showed similar features. There were large areas of interfollicular necrotising inflammation with a prominent histiocytic population and admixed lymphocytic debris, but no neutrophils (Figure 1). Immunohistochemistry revealed that the interfollicular/ paracortical areas were populated predominantly by cytotoxic/CD8-positive T-cells. The cause of death was given as mixed connective tissue disease-associated necrotising lymphadenitis and splenitis.

### Case 2

This 55 year old woman had a complex history of connective tissue disease; features of Sjogren’s syndrome and SLE were present and she also had small fibre neuropathy and asthma. She had positive anti-Ro and anti-La antibodies. She had been suffering from sciatica and was electively admitted for a decompression laminectomy of L4/L5 to alleviate her symptoms. She had an uneventful recovery and was discharged home.

10 days later, following significant sun exposure, she was re-admitted with pyrexia (40°C), dizziness, loss of balance, decreased hearing, diarrhoea, vomiting and a non-blanching rash over the upper arms and thighs. A clinical diagnosis of sepsis was made, and she was started on broad-spectrum antibiotics. No infective source was found. Stool cultures showed no evidence of Clostridium difficile and the blood and urine cultures were negative. An MRI of the lumbosacral spine showed features most in keeping with a surgical seroma. Despite treatment, her condition deteriorated over the next three days and she was admitted to ITU with lactic acidosis, haemodynamic collapse, and thrombocytopenia/DIC. Despite organ support, she died three days after her admission.

**Figure 2:**
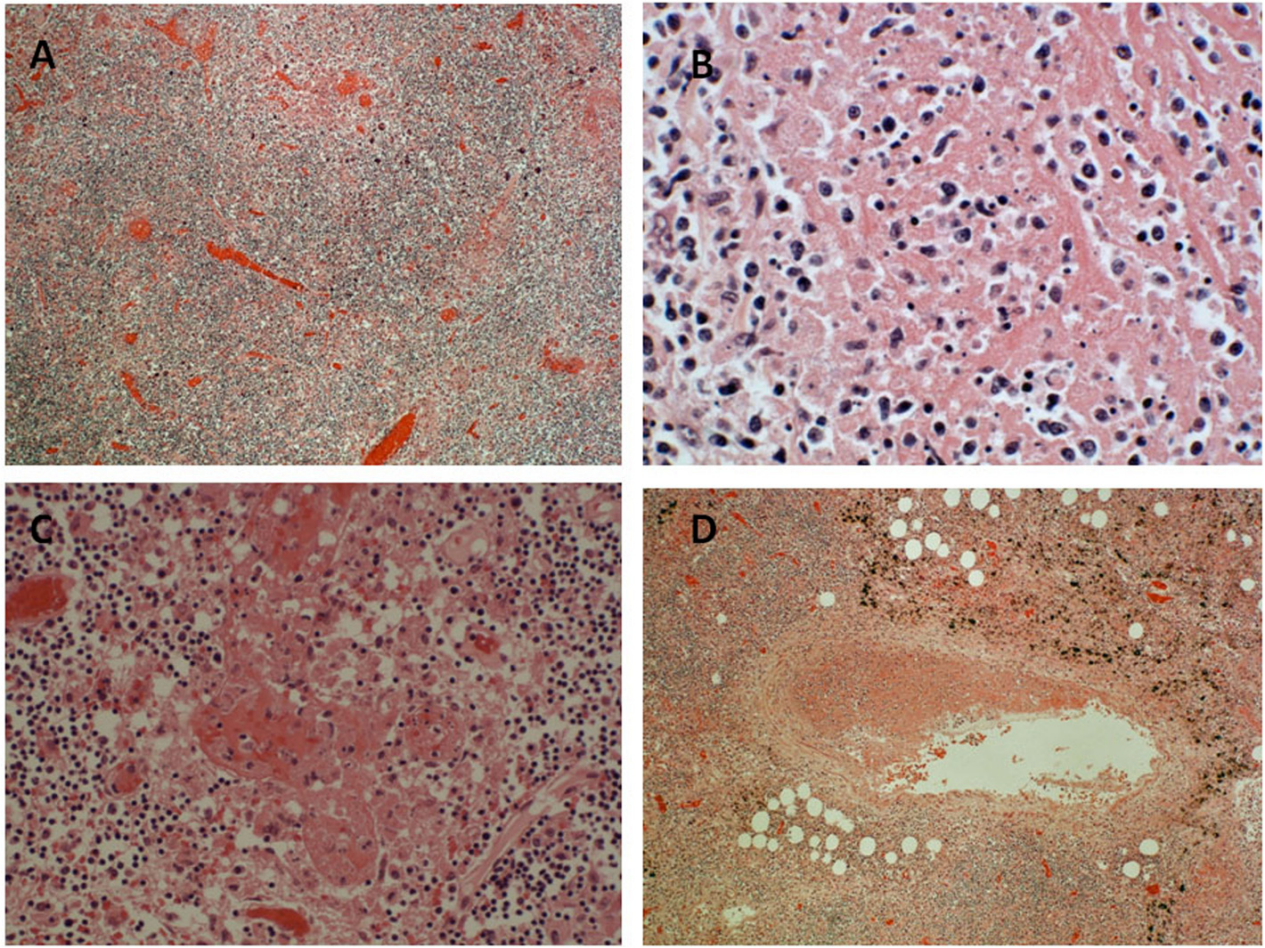
**Histology of lymph nodes from Case 2. A-B** Low (x100) and high (x400) power views of para-aortic lymph node. **C**. High power (x400) view of same lymph node showing medium vessel thrombosis. **D**. Low power (x100) view of same lymph node showing large vessel thrombosis.

**Figure 3:**
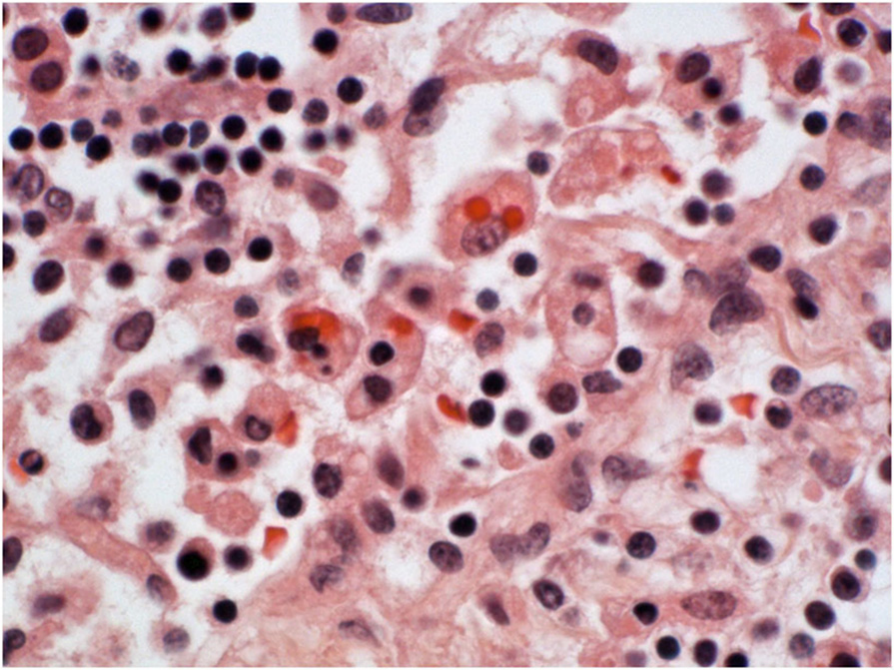
**High power (x400) view of para-aortic lymph node showing a focus of haemophagocytosis.**

Post mortem examination was unremarkable and no infective source was identified. The cervical, carinal and para-aortic lymph nodes showed paracortical necrotising inflammation with a prominent histiocytic population admixed with lymphocytes and some karryorrhectic debris, but no neutrophils (Figure 2A and B). The lymphocytes were predominantly cytotoxic/CD8-positive T-cells. The lymph nodes also showed vascular thrombosis (Figure 2C and D), and one of the para-aortic lymph nodes was extensively infarcted. Focal areas of haemophagocytosis were identified (Figure 3). The cause of death was given as SLE-associated Kikuchi disease.

## Discussion

The etiology of Kikuchi-Fujimoto disease remains unclear. Suggested causative agents which have been isolated from lymph nodes exhibiting the disease include Ebstein-Barr virus, HIV, human T-cell leukaemia virus type 1, human herpesvirus type 6 and 8, hepatitis B virus, parvovirus B19, herpes simplex, varicella zoster, cytomegalovirus, *Toxoplasma gondii*, Brucella, *Yersinia enterolitica* and parainfluenza virus (Kucukardali et al. 2007; Bosch et al. 2004; Bosch and Guilabert 2006). It has been proposed that these agents activate CD8-positive T cells leading to T-cell proliferation and apoptosis mediated by the Fas and perforin pathways. Engulfment of the apoptotic debris by macrophages would then give rise to the typical histological features. However, there is no conclusive evidence that any of these agents are the cause of Kikuchi-Fujimoto disease. In many cases, no infectious agent is found (Kucukardali et al. 2007; Bosch et al. 2004; Bosch and Guilabert 2006).

The other main proposed aetiology is autoimmune, based on the observation that a Kikuchi-like lymphadenitis occurs in the context of autoimmune disease, particularly SLE. The pathogenesis of SLE is believed to be related to defective processing of apoptotic debris, resulting in components of the debris being mistakenly presented to the immune system. Situations in which apoptosis is increased, such as Kikuchi-Fujimoto disease, could conceivably accelerate the generation of autoantibodies and increase the levels of autoantigens present, precipitating a flare-up of the disease (Santana et al. 2005). It has been suggested that the trigger may be an autoimmune response to an epithelial antigen, such as those seen in cutaneous SLE reactions (Gordon et al. 2009). Electron microscopy has shown that the histiocytes, activated lymphocytes and endothelial cells in the affected lymph nodes of Kikuchi-Fujimoto disease contain tubuloreticular structures similar to those seen in endothelial cells and lymphocytes of patients with SLE (Imamura et al. 1982). The significance of this observation is uncertain, although it hints at a common pathogenesis. Some authors consider it to be supporting evidence for a hyperimmune response of viral aetiology (Gionanlis et al. 2009). It has been suggested that Kikuchi-Fujimoto disease lies on the same disease spectrum as SLE, representing a milder form of the disease (Gionanlis et al. 2009). There is one report of two twin sisters who were human-leucocyte antigen-identical, each of whom developed Kikuchi-Fujimoto disease 10 years apart. This observation hints at the possibility of a genetic predisposition. However, neither of the sisters developed SLE or any other autoimmune sequelae (Amir et al. 2002).

Kikuchi-Fujimoto disease is a histopathological diagnosis and is probably under-recognised. It is characterised by focal cortical and paracortical necrosis with marked karyorrhexis and an infiltrate of crescentic histiocytes and plasmacytoid monocytes which lacks neutrophils. The appearances of lupus lymphadenitis can be similar. Additional features seen in lupus lymphadenitis include neutrophils, plasma cells and haematoxylin bodies, but distinction between the two entities is not always possible. Many authors recommend long-term follow-up of patients with Kikuchi-Fujimoto disease to watch for the development of SLE (Santana et al. 2005; Bosch et al. 2004; Bosch and Guilabert 2006). In the analysis of 244 reported cases of Kikuchi-Fujimoto disease by Kucukardali et al., 56 (23%) were associated non-infectious inflammatory diseases of which 32 (13%) were associated with SLE (Kucukardali et al. 2007).

In the two cases presented here, the presence of Kikuchi-like lymphadenitis in the context of connective tissue disease produced a sepsis-like clinical picture, with thrombocytopenia and DIC, which proved fatal. The differential diagnosis included true sepsis, which was the working diagnosis in both cases, and this posed a difficult therapeutic dilemma given that the true diagnoses in these cases are extremely rare, whereas sepsis is common. In our review of the literature, we found reports of 8 cases of similar life-threatening autoimmune sequelae, 2 of which were fatal, 1 of which eventually resulted in a persistent vegetative state, and 5 of which were successfully treated with pulse corticosteroids with or without cyclophosphamide (Tables 2, 3 and 4). Commencement of such therapy in these cases would have required definitive exclusion of an infective cause, for which there was not enough time due to the rapidity of the clinical decline. Tables 1 and 5 compare the clinical, biochemical and haematological features of the two cases. Both had connective tissue disease which was difficult to classify, although it was eventually felt that case 2 probably had SLE. The autoantibody profiles were different, but both were non-specific. In case 1, the necrotising lymphadenitis also involved the spleen. This is an extremely rare finding in Kikuchi lymphadenitis, and was a feature of one of the two previously reported fatal cases of SLE-associated Kikuchi lymphadenitis (Quintas-Cardama et al. 2003).

**Table 2:** **Clinical characteristics of previously reported cases of Kikuchi-Fujimoto disease occurring simultaneously with connective tissue disease**

**Necrotising lymphadenitis occurring simultaneously with connective tissue disease**				
**Country and reference**	**Age and gender**	**Connective tissue disease**	**Autoantibodies**	**Outcome**
Greece (Gionanlis et al. 2009)	23 year old Female	SLE	ANA, anti-dsDNA, Anti-Ro, Anti-La, anti-RNP	Renal failure.TTP. Responded to immunosuppression + rituximab.
USA (Smith and Petri 2013)	27 year old Female	SLE	ANA, anti dsDNA, anticardiolipin IgG and IgM, anti-Smith, anti-Ro, anti-La.	Developed lupus nephritis. Condition stable.
Japan (Aota et al. 2009)	36 year old Male	SLE	ANA	Lymphadenitis resolved with corticosteroid therapy. Developed lupus nephritis. Condition stable at 4 month follow up.
France (Gallien et al. 2008)	47 year old Male	SLE	ANA, anti-dsDNA	Lymphadenitis resolved with corticosteroid therapy.
France (Frikha et al. 2008)	14 year old Female	SLE	ANA, anti-RNP, anti-Ro	Lymphadenitis resolved with corticosteroid therapy.
France (Frikha et al. 2008)	23 year old Female	SLE	ANA, anti-dsDNA, anti-Sm, anti-RNP, anti-Ro, anti-La.	Lymphadenitis resolved with corticosteroid therapy.
Germany (Hoffmann et al. 1991)	37 year old Male	SLE	HHV-6	No life-threatening sequelae.
England (Shusang et al. 2008)	20 year old Female	SLE Had 3 year history of autoimmune hepatitis	ANA (at onset of autoimmune hepatitis) Anti-Ro, anticardiolipin (at onset of SLE)	Lymphadenitis resolved with corticosteroid therapy.
Turkey (Yilmaz et al. 2006)	53 year old Female	SLE	ANA	No life-threatening sequelae.
Singapore (Chua et al. 1996)	9 year old Female	SLE with erythema multiforme	ANA, anti-dsDNA	No life-threatening sequelae.
USA (Eisner et al. 1996)	Young male	SLE	ANA	Concurrent lupus nephritis. Complete remission with corticosteroid therapy and cyclophosphamide.
France (Leyral et al. 2005)	26 year old Female	SLE	ANA, anti-dsDNA	Haemophagocytic syndrome with EBV infection. Complete remission with corticosteroids.
Romania (Tanasescu et al. 2003)	17 year old Female	SLE and autoimmune hepatitis	Anti-Sm	Complete remission with corticosteroid therapy
Brazil (Santana et al. 2005)	20 year old Female	SLE	ANA, anti-Ro, anticardiolipin	Lymphadenitis resolved with corticosteroid therapy.
Spain (Jimenez Saenz et al. 2001)	30 year old Female	SLE	ANA, anti-dsDNA, anti-RNP, anti-scl 70	Complete remission with corticosteroid therapy.
USA (Mahajan et al. 2007)	56 year old Female	SLE	ANA, anti-dsDNA	Partial improvement with hydroxychloroquine, followed by complete remission.
USA (Gordon et al. 2009)	33 year old Male	SLE	ANA, anti-Smith, anti-RNP, anti-Ro.	Complete remission with corticosteroid therapy.
Germany (Cramer et al. 2010)	33 year old Male	SLE	ANA, anti-dsDNA	Clinical improvement with corticosteroid and mycophenolate therapy.
USA (Quintas-Cardama et al. 2003)	38 year old Female	SLE	ANA	Severe auto-immune-related clinical sequelae and cardiogenic shock. Patient died despite pulse corticosteroid and cyclophosphamide therapy.
Spain (Sopena et al. 2012)	27 year old Female	SLE	ANA	Good response to therapy.
Spain (Diez-Morrondo et al. 2012)	22 year old Female	SLE	ANA	Good response to hydroxychloroquine.
Thailand (Kampitak 2008)	50 year old Male	SLE	ANA	Developed haemophagocytic syndrome and severe hospital acquired pneumonia and septic shock. Patient died.

**Table 3:** **Clinical characteristics of reported cases of Kikuchi-Fujimoto disease occurring after the onset of connective tissue disease**

**Connective tissue disease predating necrotising lymphadenitis**				
**Country and reference**	**Age and gender**	**Connective tissue disease**	**Autoantibodies**	**Outcome**
Canada (Silver et al. 2004)	43 year old Female	Discoid lupus erythematosus for 10 years	None	Recovered without treatment
India (Londhey et al. 2010)	39 year old Female	SLE for 2 years (not initially diagnosed)	ANA and anti-dsDNA	No life-threatening sequelae. Responded to corticosteroids and hydroxychloroquine. Asymptomatic at 1 year follow-up.
Canada (Pace-Asciak et al. 2008)	35 year old Female	SLE (duration not stated)	NA	Lymphadenitis resolved with conservative management.
France (Frikha et al. 2008)	30 year old Female	Mixed connective tissue disease for 8 years	ANA, anticardiolipin	Lymphadenitis resolved with corticosteroid therapy.
Taiwan (Chen and Lan 1998)	4 cases All female Ages 21–35 years old	SLE for 8 days to 2 years	Ribosomal-P antibodies (1 case) RNP (1 case) None (2 cases)	No life-threatening sequelae
France (Abraham et al. 2011)	35 year old Female	Overlap syndrome for 12 years	Antiphospholipid antibodies	Lymphadenitis resolved with corticosteroid therapy.
Sabah (Bachi 2002)	27 year old Female	SLE – systemic symptoms for 2 months	ANA, anti-Ro, anti-La at onset of lymphadenitis	Complete remission with corticosteroid therapy.
Japan (Wano et al. 2000)	37 year old Female	SLE for 2 years	NA	Haemophagocytic syndrome. Complete remission with corticosteroid pulse therapy.
India (Londhey et al. 2010)	39 year old Female	Onset of connective tissue disease for 4 years. Lymphadenitis first appeared after 2 years. SLE diagnosis established later.	ANA, anti-dsDNA.	Complete remission with corticosteroid therapy.
Spain (Sopena et al. 2012)	2 cases 22 and 32 years old Both Female	Both SLE. Onset not stated.	ANA only (1 case) ANA, anticardiolipin, anti-Ro, RNP (1 case)	Good response to therapy
Italy (Ruaro et al. 2014)	19 year old Male	SLE, onset 2–3 months prior to diagnosis of Kikuchi lymphadenitis	ANA only	Initial good response to therapy, single episode of relapse 2 years after initial onset, subsequently symptom free on corticosteroid and methotrexate therapy.

**Table 4:** **Clinical characteristics of reported cases of Kikuchi-Fujimoto disease occurring before the onset of connective tissue disease**

**Necrotising lymphadenitis predating connective tissue disease**				
**Country and reference**	**Age, gender**	**Connective tissue disease**	**Autoantibodies**	**Outcome**
Saudi Arabia (el-Ramahi et al. 1994)	22 year old Female	SLE after 5 months	ANA, anti-Sm, anti-dsDNA, anti-Ro, anti-La.	Lymphadenitis resolved with corticosteroid therapy.
Saudi Arabia (el-Ramahi et al. 1994)	34 year old Female	SLE after several months	ANA, anti dsDNA, anti-Sm	Not stated.
Thailand (Sanpavat et al. 2006)	28 year old Female	SLE after 44 weeks	ANA, anti-dsDNA	Lymphadenitis resolved with corticosteroid therapy.
Spain (Paradela et al. 2008)	17 year old Female	SLE onset after 9 months	ANA, anti-dsDNA	No life-threatening sequelae.
Australia (Goldblatt et al. 2008)	22 year old Female	SLE after 3 months	ANA, anti-Ro, anti-Sm at onset of SLE.	Complete remission with corticosteroid therapy.
Australia (Goldblatt et al. 2008)	39 year old Female	SLE after 14 months	ANA, anti-dsDNA, anti-Ro, anti-Sm, anticardiolipin.	Complete remission with immunosuppression.
Australia (Goldblatt et al. 2008)	31 year old Female	SLE after 6 months	ANA, anti-dsDNA, anti-Ro at onset of SLE	Treated with corticosteroids, azathioprine and hydroxychloroquine. 2 years later, developed meningoencephalitis leading to a persistent neuro-vegetative state.
Australia (Goldblatt et al. 2008)	14 year old Female	SLE after a few days	Anti-dsDNA at onset of SLE.	Complex clinical course with lupus nephritis. Eventual remission with B-cell depletion therapy.
Spain (Alijotas-Reig et al. 2008)	31 year old Female	SLE after years. Lymphadenitis was pregnancy-associated and did not recur at onset of SLE.	ANA, anti-RNP, borderline anti-dsDNA at onset of SLE.	Persisting arthralgias and asthenia.
Japan (Ogata et al. 2010)	7 year old Male	Mixed connective tissue disease after 2 months	Weak anti-Ro, anti-La, anti-Sm, antiphospholipid and anti-RNP at onset of connective tissue disease	Complete remission with corticosteroid therapy.
Japan (Ogata et al. 2010)	6 year old Female	SLE after 2 years	ANA, anti-dsDNA, anti-Sm, anti-RNP at onset of SLE.	Complete remission with corticosteroid and cyclophosphamide pulse therapy.
Spain (Sopena et al. 2012)	4 cases 27–32 years old All Female	Sjogren syndrome (2 cases) SLE (1 case) SLE-like (1 case)	ANA, anti-Ro (Sjogren cases) ANA only (SLE-like case)	1 case had a near-fatal clinical course, treated with intravenous immunoglobulins. 2 of the remaining cases had relapses of lymphadenitis.
USA (Zuo et al. 2012)	23 year old Female	SLE after three months	ANA and rheumatoid factor at onset of lymphadenitis ANA, anti-Ro, anti-La, anti-dsDNA, anti-Smith, anti-RNP at onset of SLE	Not stated.
India (Patra and Bhattacharya 2013)	30 year old Female	SLE after 2 years	ANA, anti-dsDNA at onset of SLE	Complete remission with immunosuppression.
Italy (Di Lernia et al. 2013)	42 year old Female	Cutanerous lupus after two months	Anti-Ro, ENA at onset of cutaneous lupus	Complete remission with prednisolone and hydroxychloroquine.
India (Patra and Bhattacharya 2014)	30 year old Female	SLE after two years	ANA and anti-dsDNA at onset of SLE	Complete remission with immunosuppression

**Table 5:** **Comparison of the main clinical and pathological features of the two cases (DIC = disseminated intravascular coagulation; SLE = systemic lupus erythematosus)**

	**Case 1**	**Case 2**
**Age and Gender**	57 year old female.	55 year old female.
**Past Medical History**	Mixed connective tissue disease - including arthritis, Raynaud’s phenomenon and rheumatoid nodules/granuloma annulare. Psoriasis. Multiple drug allergies.	SLE/ Sjogren’s syndrome. Small fibre neuropathy. Asthma.
**Clinical presentation**	No sun exposure. Pyrexia (38-40°C), flank pain, urinary frequency, erythematous rash on trunk. Deteriorated into sepsis-like clinical picture.	Sun exposure. Pyrexia (40°C), non-blanching rash over upper arms and thighs, sepsis-like clinical picture.
**Autoantibodies**	ANA with speckled pattern, anti-La and RNP.	Anti-Ro and Anti-La.
**Thrombocytopenia/DIC**	Yes	Yes
**Acidosis**	Yes	Yes
**Lymph node histology**	Necrotising lymphadenitis.	Necrotising lymphadenitis.
**Blood vessel thrombosis**	No	Yes
**Spleen involvement**	Yes	No

Case 2 had a number of features which are more closely associated with SLE. Firstly, there was a history of sun exposure. In this context, it is noteworthy that anti-Ro and anti-La antibodies, which were positive in this patient, are particularly associated with photosensitivity (Sontheimer et al. 1979). One model of the pathogenesis of photosensitivity in SLE proposes that exposure of keratinocytes to ultraviolet light increases the surface expression of Ro antigen. Anti-Ro antibodies would then bind these receptors and precipitate a cutaneous reaction which then begets a systemic reaction (Furukawa et al. 1999). It is tempting to speculate that such a mechanism may have triggered the final illness in Case 2, but this cannot be known for certain. This case showed vascular thrombosis in the affected lymph nodes, a feature which is seen in SLE.

Case 2 also showed focal evidence of haemophagocytosis in the lymph nodes. This was an interesting observation, but one whose significance is uncertain. Haemophagocytic syndrome has been previously described in cases of Kikuchi-Fujimoto disease occurring in the context of SLE (Wano et al. 2000; Kampitak 2008; Leyral et al. 2005). However, there was no conclusive evidence for the presence of the haemophagocytic syndrome in this case.

In our review of the literature, we found 55 cases of Kikuchi-Fujimoto disease occurring in the context of definite connective tissue disease, 50 of which were associated with SLE. Of the 55 cases, 22 (40%) had simultaneous onset with, 19 (35%) predated the onset of and 14 (25%) developed after the associated connective tissue disease. The association was usually associated with a flare-up of disease activity, the severity of which varied. Life-threatening autoimmune sequelae, as discussed above, were reported in 8 cases, 2 of which resulted in fatalities (Tables 2, 3 and 4).

The appearance of autoantibodies was associated with the onset of the connective tissue disease rather than the lymphadenitis in all but 2 cases. Of the two cases in which autoantibodies were linked to the onset of lymphadenitis, one developed further antibodies following the onset of SLE (Bachi 2002; Zuo et al. 2012). There were several reported cases of Kikuchi-Fujimoto disease occurring in the context of skin manifestations, which provided interesting but conflicting insights into the relationship between the lymphadenitis and the appearance of autoantibodies (Prignano et al. 2012; Paradela et al. 2008; Yilmaz et al. 2006; Mootsikapun et al. 2002; Silver et al. 2004). On the one hand, two of these cases had positive ANA antibodies, but a diagnosis of SLE was excluded (Prignano et al. 2012; Yilmaz et al. 2006). On the other hand, one was a case of discoid lupus who developed Kikuchi-Fujimoto disease without developing autoantibodies (Silver et al. 2004), and another was a case which went on to develop SLE, with auto-antibodies appearing only at the onset of SLE (Paradela et al. 2008). As an aside, the authors of this report made the observation that cases of cutaneous manifestations of Kikuchi-Fujimoto disease which progressed to SLE were associated with interface dermatitis, and suggested that this feature may be a predictor of progression. In one case, the lymphadenitis preceded the appearance of cutaneous lupus, with autoantibodies appearing only at the onset of cutaneous lupus (Vassilakopoulos et al. 2010). Autoantibodies have also rarely been reported to appear in the classical self-limiting form of Kikuchi’s lymphadenitis, resolving when the lymphadenitis resolves (Vassilakopoulos et al. 2010). Finally, one intriguing report described a case of Kikuchi-Fujimoto disease and systemic SLE-like symptoms without any autoantibodies. The symptoms resolved spontaneously within 3 months without treatment, and did not recur (Mootsikapun et al. 2002). Overall, although Kikuchi-like lymphadenitis can rarely be associated with the appearance of autoantibodies, these cases would appear to be the exception rather than the rule. This observation would argue against a straightforward autoimmune aetiology.

In conclusion, Kikuchi-Fujimoto disease occurring in the context of connective tissue disease does not usually take the benign self-limiting course which typifies the classical form of the disease. It is usually associated with a flare-up of the connective tissue disease requiring treatment, and can lead to severe, potentially life-threatening sequelae. The aetiology of this effect is unclear, but clinically it can mimic sepsis. In this context, the possibility of auto-immune sequelae in patients with known autoimmune disease should always be considered if no infective source is identified. Although the classical self-limiting form of Kikuchi-Fujimoto disease appears to represent a distinct clinical and pathological disease, it is unclear whether it is always the same entity, regardless of the context in which it occurs, or whether it represents a histological pattern with a variety of possible causes.
